# Dimensionality-modulated generative AI for safe biomedical dataset augmentation

**DOI:** 10.1016/j.isci.2025.114321

**Published:** 2025-12-02

**Authors:** Jörn Lötsch, André Himmelspach, Dario Kringel

**Affiliations:** 1 Goethe University, Institute of Clinical Pharmacology, Faculty of Medicine, Theodor-Stern-Kai 7, 60590 Frankfurt am Main, Germany; 2 University of Helsinki, Faculty of Medicine, Haartmaninkatu 8, P.O. Box 63, 00014 Helsinki, Finland; 3 Fraunhofer Institute for Translational Medicine and Pharmacology (ITMP), Theodor-Stern-Kai 7, 60596 Frankfurt am Main, Germany

**Keywords:** Artificial intelligence, Health informatics

## Abstract

Generative AI can expand small biomedical datasets but may amplify noise and distort statistical relationships. We developed genESOM, a framework integrating an error control system into a generative AI method based on emergent self-organizing maps. By separating structure learning from data synthesis, genESOM enables dimensionality modulation and injection of engineered diagnostic features, i.e., permuted versions of real variables, as negative controls that track feature importance stability. A data-driven stopping criterion halts augmentation when error inflation begins, limiting overfitting. Validation across two artificial and six biomedical datasets, including preclinical and clinical domains, showed that moderate augmentation (one synthetic sample per original) preserved variable ranking and yielded strong negative correlations (Kendall’s tau: −0.53 to −0.85) between statistical significance and feature selection frequency. Excessive augmentation disrupted these relationships. Moderate preclinical augmentation safely doubled sample sizes without compromising analytical reliability and supported up to 50% reductions in laboratory animal use.

## Highlights

• Dimensionality modulation in generative AI genESOM enhances error control
• Engineered control features serve as error detection signals in genAI
• Safe augmentation could reduce laboratory animal use by up to 50%

## Graphical abstract

**Figure:**
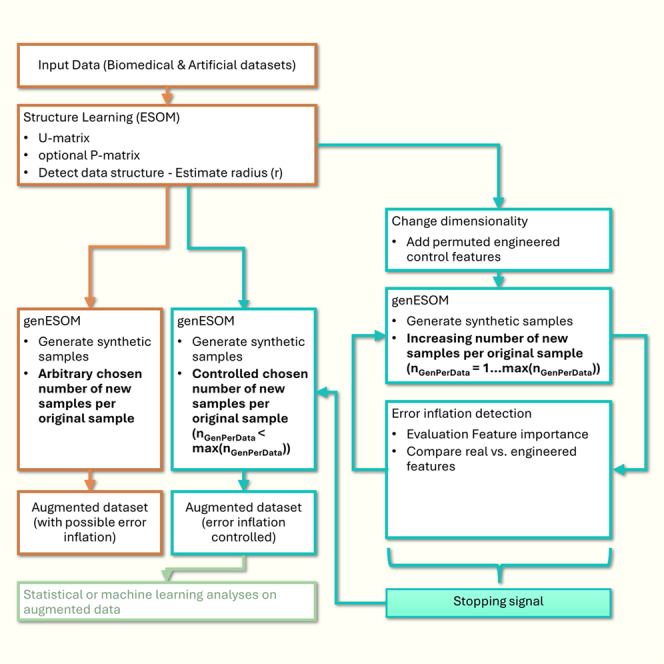

## Introduction

Generative AI (genAI) synthesizes new data that mirror patterns in the training set. This process typically unfolds in two stages: first, a structure learner identifies latent features in the data through probabilistic inference; second, a generator uses these learned structures to create synthetic samples. In formal terms, the goals of generative learning are (1) to estimate the joint distribution and (2) to generate new data instances and associated class labels.^1^ Generative models are thus well-suited for augmenting small datasets, which is a recurring challenge in biomedical and preclinical research where limited patient cohorts, scarce biosamples, or restrictions on animal experiments reduce statistical power, hinder reproducibility, and slow translation of findings to clinical advances.^2^^,^^3^^,^^4^ Increasing sample sizes through *in silico* augmentation is, therefore, an attractive solution.

However, generative algorithms can also propagate errors. As with conventional oversampling techniques,^5^ genAI may amplify stochastic noise, inflate type I error rates,^6^^,^^7^ and even exacerbate drift when trained iteratively on self-generated data.^8^ Current oversampling approaches (e.g., SMOTE, random noise injection) suffer similar risks, producing spurious correlations and unreliable results.^9^ The absence of safe, practical generative AI tools with built-in safeguards against false-positives represents a critical gap in biomedical research, where irreproducibility already imposes substantial financial burdens and undermines confidence in reported findings. Even modest improvements in error control could yield significant financial and translational benefits: the preclinical Contract Research Organization (CRO) market alone accounts for tens of billions of USD annually and is projected to double in size (https://straitsresearch.com/report/preclinical-cro-market), underscoring the potential return on investment for reliable augmentation frameworks. Indeed, attempts at AI use in this research environment are under way.^10^^,^^11^ However, the practical adoption of generative AI for biomedical dataset augmentation remains constrained by intrinsic error inflation behavior, an unavoidable liability that currently limits its safe deployment in preclinical research contexts.

**Figure 1:**
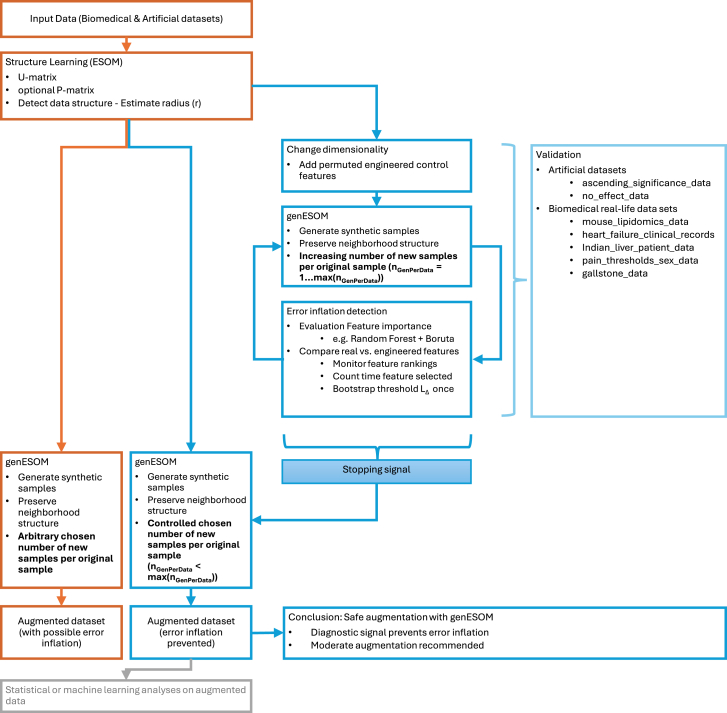
Flowchart illustrating the main steps of the present approach The orange section (left) represents the previously published genESOM data generation process,^12^ which is included here only for context and is neither re-evaluated nor benchmarked again in this report. The blue sections (right) depict the novel components introduced in this work, namely, the error inflation control mechanism and its integration into genESOM. Below the generated augmented dataset, possible downstream analyses are indicated schematically as examples of potential applications. These analyses, as well as prior validation experiments with machine learning classifiers, are beyond the scope of the present report and are included only to provide context for how the method may be applied.

Therefore, in this work, we present a generative AI framework that explicitly addresses this limitation by integrating error diagnostics within a recently developed model based on emergent self-organizing maps (genESOM)^12^ (blue parts in Figure 1). Unlike most generative models, genESOM separates structure learning from data generation, enabling dimensionality modulation between phases. This unique property allows the injection of engineered diagnostic signals, constructed as permuted counterparts of original variables, serving as built-in controls for error inflation. By tracking their influence during data augmentation, the method provides an implicit, data-driven stopping criterion to prevent overfitting. Synthetic samples are generated under a structure-informed neighborhood radius, preserving intrinsic topology, while avoiding artifacts of jitter-based oversampling. Designed for limited-sample contexts, including preclinical, clinical, and laboratory studies, genESOM not only addresses a major methodological gap but also offers considerable translational and economic potential by reducing error inflation and improving reproducibility across the biomedical research ecosystem. The present error inflation control framework is a prerequisite for leveraging generative AI to enhance preclinical and clinical research, particularly in settings where small sample sizes pose significant challenges.

To monitor error inflation resulting from the addition of AI-generated data, we introduce engineered features as control signals. These features are irrelevant to the class structure of the dataset. Engineered from the original features, they are added to the dataset after the initial data structure detection step and before the generative step. This prevents the engineered additional features from influencing the detection of the structure of the dataset. However, they are subjected to augmentation as the “true” variables, which includes that they are similarly affected by the inflation of random effects and errors. They can thus be used to identify when the results of the generative process start to become unreliable. This point is reached when these features, which are known to be class-structurally irrelevant, start overtaking true features of the dataset in the ranking of variables for feature importance.

We will show that this is a progressive effect increasing with the number of data points generated relative to the original sample size. Therefore, data generation can and must stop when these engineered signal features are falsely selected as class-structure-relevant features. Similarly, when augmenting a dataset with a predefined number of data points to generate, these engineered signal features can serve as control markers to ensure that the results obtained on the augmented data are valid and do not present an alpha error inflation artifact.

The present method is conceived as an important add-on to the previously published genESOM framework, providing a crucial prerequisite for applying genESOM in research practice with small datasets without risking invalid results due to alpha error inflation. The scope of this report is, therefore, limited to this new component and does not include a re-evaluation of genESOM itself, such as comparisons with alternative generative AI methods, downstream analyses of augmented data, or repetition of previously reported classifier-based discrimination experiments. In those earlier experiments, widely used machine learning classifiers (random forests and support vector machines) were unable to distinguish generated from original data better than chance, thereby underscoring the validity of synthetic data generation by genESOM.^12^ Here, genESOM is briefly recapitulated only to facilitate understanding of the new error inflation control mechanism that constitutes the focus of this report. A schematic presentation of the overall workflow, indicating which components are the focus of this report, is provided in Figure 1.

## Results

The generative ESOM method has been detailed elsewhere, including validations on multiple artificial and real-world datasets.^12^ In essence, it consists of two sequential components: a structure-detection ESOM of artificial neurons for distance- and density-based pattern discovery, using the classical ESOM/U-matrix approach,^13^^,^^14^ followed by a generative module (genESOM) that was recently introduced.^12^ A key parameter derived from the first component guides data generation for dataset augmentation, with further technical details provided in the STAR Methods section of this report.

### Structure-preserving data augmentation is associated with amplification of random noise

#### Structure-preserving data augmentation

**Figure 2:**
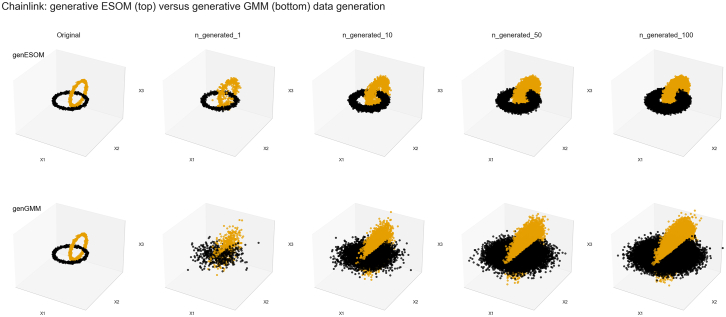
Visualization of augmentation of the three-dimensional Chainlink artificial dataset^16^ The synthetic dataset consists of *n* = 1,000 data points evenly distributed over k = 2 classes (color coded) and arranged in the form of two intertwined rings. The top row of panels shows the results of genESOM-based data generation. Starting with the original dataset (no generation), it includes an increasing number of generated data points per original data point from left to right. The bottom row shows the results of the same task when a generative Gaussian mixture model (genGMM)-based approach was used (compare Ultsch and Lötsch^12^ for further examples of data generation using genESOM and alternatives).

Structure-preserving data augmentation has been demonstrated through various examples in Ultsch and Lötsch.^12^ For the present purpose, we recur to an example that was taken from the so-called Fundamental Clustering Problems Suite (FCPS), freely available at https://www.mdpi.com/2306-5729/5/1/13/s1 or in the R library FCPS (https://cran.r-project.org/package=FCPS^15^). The FCPS is a collection of small artificial datasets designed to address specific problems of structure discovery in high-dimensional spaces. The three-dimensional “Chainlink” dataset consists of two classes, each with 500 instances, sampled uniformly from a torus with minor and major radii of *r* = 0.1 and *R* = 1, respectively.^12^ The two tori are intertwined orthogonally with the maximum possible distance between them (Figure 2, left panels).

The Chainlink dataset was processed using the genESOM data augmentation pipeline (see above) and our R package “Umatrix” (https://cran.r-project.org/package=Umatrix^17^). First, the data structure was analyzed using ESOM and the U-matrix. Then, the radius parameter r was determined. Then, synthetic data points were generated in varying amounts per original data point (i.e., 1, 10, 50, and 100 generated points per original point).

For comparison, new instances for the Chainlink dataset were also generated using a Gaussian mixture model (GMM)^18^ as a probabilistic model that assumes that all data points are generated from a mixture of (presently, *m* = 2) Gaussian distributions, whose parameters are initially unknown. The Expectation-Maximization (EM) algorithm was used to optimize the maximum likelihood of the GMM model, as implemented in the R package “Mclust” (https://cran.r-project.org/package=mclust^19^).

The generative algorithms produced data resembling the original structure (Figure 2). As previously observed in Ultsch and Lötsch,^12^ genESOM generated new data that effectively augmented the existing two-ring structure of the Chainlink dataset. We reproduce this experiment to illustrate genESOM’s core method, allowing readers to understand it without consulting the original publication. In contrast, the new data produced by the GMM approach preserved the original dataset structure less clearly as observed previously.^12^ These results demonstrate that genESOM is capable of maintaining the structural integrity of a dataset. genESOM has also been extensively compared to alternatives such as the generative use of GMM (genGMM), as well as other methods like autoencoders or Tabular Generative Adversarial Network (TGAN), previously evaluated on the same dataset (see figure 14 in Ultsch and Lötsch^12^).

#### Amplification of random noise and inflation of the statistical alpha error

Generating new data based on the learned structure of existing data carries an inherent risk of overfitting or inflating type I error rates (alpha error) by amplifying small, random variations present in the original dataset.^6^^,^^20^ To investigate this effect, the above experiment was extended with statistical testing.

**Figure 3:**
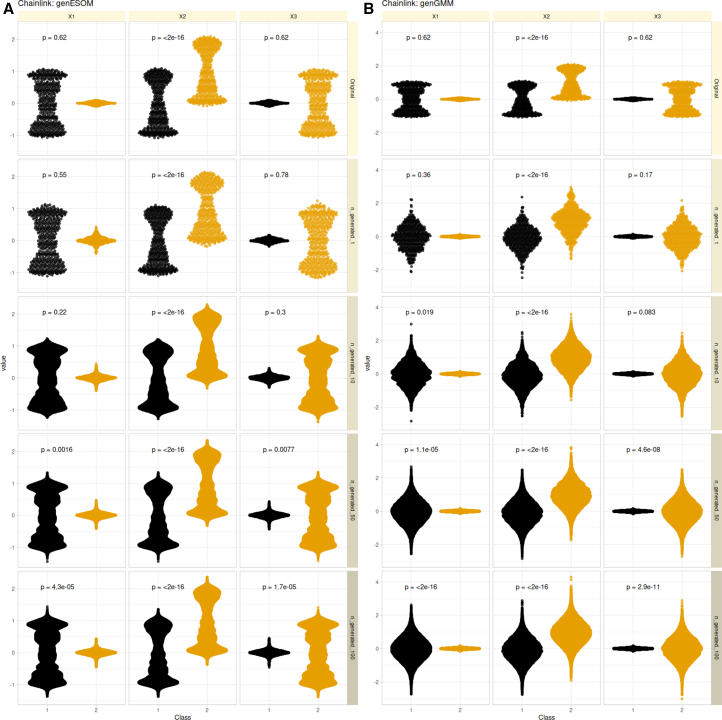
Alpha error inflation is a side effect of data generation by generative algorithms Exemplified with two algorithms: the currently addressed genESOM and, as a suitable but not exhaustive competition, Gaussian mixture models (genGMM).The three-dimensional Chainlink artificial dataset^16^ consists of 1,000 data points evenly distributed over two classes (color coded) and arranged in the form of two intertwined rings. As the number of data points generated per original data point increases (from 0 to 1, 10, 50, and 100), the significance levels of the class differences increase successively. The boxplots show the individual data points arranged to display the density distribution of the three variables. (A) Results of genESOM-based data generation. (B) Results of genetic data generation using genGMM. The *p* values are derived from univariate *t* test comparisons of the variable between classes, without applying α correction.

In the original two-class Chainlink dataset, only the second variable (“X2”) happens to differ statistically significantly between the two classes (Figure 3, top panels). However, as more synthetic data instances were generated, the degree of significance of the differences between classes increased successively (Figure 3, second top to bottom row of panels). This effect was more pronounced with data generated by the genGMM approach than by the genESOM approach. While this observation is anecdotal, along with the observations shown in Figure 2, it suggests that compared to genESOM, genGMM produces data that fit less well into the original data structure and are more susceptible to alpha error inflation. However, the experiment clearly demonstrates that genESOM is also susceptible to the amplification of random noise and accidental differences in the data.

### Development of an inherently implementable error inflation control mechanism

The proposed error inflation control mechanism proceeds in three steps. First, the ESOM method, as described above, is employed to learn the intrinsic structure of the dataset. Second, engineered control features are incorporated to provide a diagnostic baseline, enabling quantification of error inflation. Third, synthetic data points are generated using the learned structure in the first step, with augmentation governed by the error control variables from the second step.

#### Use of ESOM to learn data structure and dimensionality adjustment

In the first step, the generic ESOM algorithm is used to learn the data-specific critical radius *r*. ESOM allows for flexible adjustment of the dataset’s dimensionality. Specifically, after learning the structure of some Dataset *D* with dimensionality *J*, it is possible to generate data points not only for *D* but also for an extended Dataset *D*′,

(Equation 1) $$D=\left\{X_{1},\ldots ,X_{J}\right\}\;\to D^{\prime }=\left\{X_{1},\ldots ,X_{J},X_{J+1},\ldots ,X_{j+d}\right\},d\geq 0\text{.}$$

Such adaptability is unique to genESOM and forms the basis of the current error control strategy. By decoupling the learning of data structure from the generation of new samples, genESOM enables principled control over the complexity and variability of datasets.

#### Engineered control features in generative AI data augmentation

This decoupling allows us to insert artificial control features for the second step. These features are deliberately constructed and incorporated into the dataset prior to generative augmentation, ensuring they are processed in parallel with the original variables but only after the intrinsic structure of the data has been learned, thereby preventing contamination of the structural estimation. Serving as negative controls, engineered features allow for unbiased assessment of feature selection and importance, providing a built-in diagnostic for evaluating the reliability of results derived from generative model-based research. Specifically, for each original variable *X*_*j*_, a permuted counterpart $X_{j}^{perm}$ is generated by randomly shuffling its values across samples,

(Equation 2) $$X_{j}^{perm}=Permute\left(X_{j}\right)\text{,}$$

where *Permute*(·) denotes a random permutation operator applied independently to each variable. This process results in a set of control features that retain the marginal distribution of the original variables but lack any genuine association with the target or other predictors.

To assess when data generation should be stopped, feature importance is computed for all variables to evaluate the informativeness of each original feature relative to its diagnostic counterpart by computing the difference in their importance scores,

(Equation 3) $$\Delta I_{j}=I\left(X_{j}\right)-I\left(X_{j}^{perm}\right)\text{,}$$

where *I*(·) denotes the feature importance assigned by the model. A consistently positive Δ*I*_*j*_ indicates that the original feature carries genuine predictive value beyond what can be explained by random structure, while Δ*I*_*j*_ values near zero or negative suggest spurious or non-informative features.

#### Detection of error inflation as data generation stopping signal

To prevent excessive data augmentation as indicated by Δ*I*_*j*_, a principled threshold must be established to indicate when exactly augmentation should stop.

To robustly estimate this threshold for error inflation in feature importance, we employ a bootstrap resampling approach. Let the number of bootstrap samples be *n*_*Bootstrap*_ = 100,000. Define vector *a* as the selection counts of permuted variables across bootstrap samples and vector *b* as the selection counts of the original (true) variables across bootstrap samples. For each bootstrap iteration, samples $a_{sample}=\left\{a_{1},a_{2},\ldots ,a_{n_{Bootstrap}}\right\}$ and $b_{sample}=\left\{b_{1},b_{2},\ldots ,b_{n_{Bootstrap}}\right\}$ are drawn with replacement from *a* and *b*, respectively.

The bootstrap difference vector between original and permuted variable selections is computed as Δ_*Bootstrap*_ = *b*_*sample*_-*a*_*sample*_ = {*b*_*i*_-*a*_*i*_|*i* = 1, …,*n*_*Bootstrap*_}. The limit of validity for error inflation detection, *L*_Δ_, is then defined as the 95^th^ percentile of this difference distribution,

(Equation 4) $$L_{\Delta }=Q_{0.95}\left(\Delta_{Bootstrap}\right)\text{.}$$

After computing *L*_Δ_, the base genESOM algorithm can be used to successively augment the dataset by generating $n_{GenPerData}\in N_{>0}$ synthetic data points per original one. Both the original and permuted variables are subjected to the same generative augmentation procedures using the previously obtained critical radius *r* applied to all original and control variables with new data points inheriting the class of the original data point. The number of generated samples is increased stepwise until the selection frequency Δ*I*_*j*_ exceeds the threshold *L*_Δ_. The stopping criterion is then defined as the largest *n*_*GenPerData*_ for which the threshold is not yet surpassed.

#### Generation of data with stopping criterion

After computing the stopping criterion *n*_*GenPerData*_, the original dataset *D* can now be augmented with *n*_*GenPerData*_ new data points per original one safely, as the synthetic samples are produced only within the bounds of statistical validity. This provides a principled safeguard against inflated feature importance and false discoveries. A complete overview of our proposed method in the form of a pseudocode is given in Box 1.

Box 1 **Model-agnostic pseudocode of the framework of genESOM with error inflation detection**

 **Input**: *D* = {*X*_1_, … ,*X*_*J*_,*Y*}, a Dataset with variables *X*_*J*_ and target *Y*

1: Use ESOM algorithm on *D* to obtain critical radius *r*

2: *D*_*temp*_←*D*

3: Add permuted variable $X_{j}^{perm}$ for every original variable *X*_*j*_ into *D*_*temp*_

4: *a*,*b*←∅

5: **for** *i*∈{ 1, … ,*n*_*Bootstrap*_} **do**

6:  Apply feature selection method to *D*_*temp*_

7:  Insert number of times a permuted variable has been selected into *a*

8:  Insert number of times an original variable has been selected into *b*

9: **end for**

10: Δ_*Bootstrap*_←{*b*_*i*_-*a*_*i*_|*i* = 1, …,*n*_*Bootstrap*_}

11: Set error threshold as *L*_Δ_←*Q*_0.95_(Δ_*Bootstrap*_)

12: Set stopping signal *n*_*GenPerData*_←1

13: **while** TRUE **do**

14:  Generate *n*_*GenPerData*_ amount of new data points for every original one using *r* for all *X*_*j*_ and $X_{j}^{perm}$ in *D*_*temp*_, while inheriting new *Y* value from original data point

15:  Add new data points to *D*_*temp*_

16:  Apply feature selection to *D*_*temp*_ to obtain feature importance *I*

17:  Calculate $\Delta I_{j}\leftarrow I\left(X_{j}\right)-I\left(X_{j}^{perm}\right)$ for every original variable *X*_*j*_ and its permuted counterpart $X_{j}^{perm}$

18:  **if** Δ*I*_*j*_>*L*_Δ_ for any *j* **then** BREAK

19:  *n*_*GenPerData*_←*n*_*GenPerData*_+1

20:  Reset *D*_*temp*_ by removing newly added data points from *D*_*temp*_

21: **end while**

22: Generate *n*_*GenPerData*_ amount of new data points for every original one using *r* for all *X*_*j*_ in *D*, while inheriting new *Y* from original data point

23: Add new data points to *D*

24: **return** *D*

#### Complexity

The computational complexity of the proposed approach can be assessed by its three main steps. First, the ESOM algorithm requires identifying the Best Matching Unit (BMU) for each of the *n* data points among *m* neurons over a total of *E* epoch times, leading to a complexity of *O*(*E*·*n*·*m*). In addition, a Gabriel graph *G* and a Gaussian mixture model must be fitted to determine the critical radius *r*. Constructing the Gabriel graph over all BMU’s has a cost of *O*(*m*·*logm*).^21^ Fitting the Gaussian mixture model introduces in its standard implementation an additional cost of *O*(*n*· 2 ·*J*^3^).^22^ Second, the bootstrapping procedure involves applying a feature importance algorithm, such as Boruta (see STAR Methods), *n*_*Bootstrap*_ number of times and then calculating the stopping criterion by generating synthetic data point using the critical radius *r* until the bootstrapping threshold is reached in *T* amount of tries. This requires *O*(*T*·*n*·*n*_*GenPerData*_·*J*) operations, since for each of the *n* original data points, *n*_*GenPerData*_ new points are generated across all *J* variables. Third, the determined stopping criterion *n*_*GenPerData*_ is used to generate the data points one last time, which again takes *O*(*n*·*n*_*GenPerData*_·*J*). If Boruta is used as the feature selection method with a complexity of *O*(*n*·*J*),^23^ this leads to a total complexity of *O*(*E*·*n*·*m*)+*O*(*m*·*logm*)+*O*(*n*· 2 ·*J*^3^)+*O*(*n*_*Bootsrap*_·*n*·*J*)+*O*(*T*·*n*·*n*_*GenPerData*_·*J*)+*O*(*n*·*n*_*GenPerData*_·*J*). As in practice *n*_*Bootsrap*_≫*n*,*J*,*E*,*n*_*GenPerData*_,*T*, the complexity is mainly dominated by bootstrapping step. However, a high number of variables can also substantially increase computational cost.

### Preservation of variable importance ordering in the ascending significance dataset

**Figure 4:**
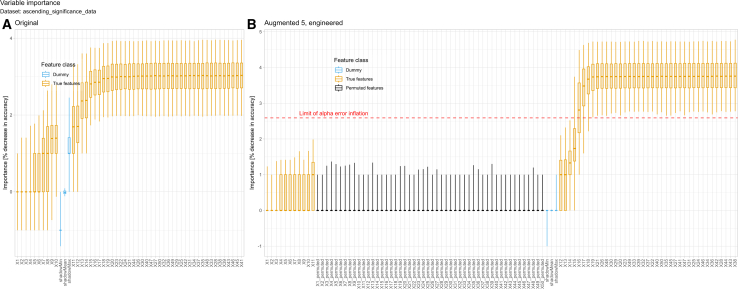
Variable importance distributions in the artificial dataset with ascending statistical significance of class differences (“ascending_significance_data”) for the original and augmented (5×) data regimes The variable importance distributions are shown for the original and augmented (5×) data regimes. The boxplots show the distribution of feature importance scores (as measured by the Boruta algorithm) for each variable in the original dataset (left) and the dataset augmented with five synthetic samples per original observation (right). For each variable, the central line indicates the median importance, the box spans the 25^th^ to 75^th^ percentiles, and the whiskers extend to the 2.5^th^ and 97.5^th^ percentiles, thus representing the central 95% of observed importance values across iterations. True features, engineered features, and Boruta dummy features are distinguished by color. The dashed horizontal line marks the empirical upper limit of importance expected for non-informative features. This plot shows that the most informative variables are clearly different from engineered and dummy features and that the method can distinguish them even after substantial data augmentation.

Using an artificial dataset with ascending statistical significance of class differences (“ascending_significance_data”), the variables with the smallest class differences were ranked lower than the shadow features employed by the Boruta feature selection method, while the variables with the largest class differences were ranked higher (Figure 4). After introducing non-informative features, all of them were consistently ranked below the original variables. The original variables were selected as important much more frequently than the engineered features across all data regimes. This pattern confirms that the method effectively distinguishes between informative and non-informative variables and that the error inflation control operates as intended.

The order of variable importance closely mirrored the systematic increase in class differences across variables, and it remained stable when the dataset was augmented with synthetic samples (Figure 4). The order of feature importance basically paralleled the succession of *p* values obtained from *t* tests across variables, with those exhibiting larger true class differences consistently identified as statistically significant in the sequence of decreasing *p* values. Even at high levels of augmentation (50 synthetic samples per original), the original signal structure remained dominant, and the proportionality between class difference size and feature selection frequency was largely maintained. Only minor deviations were observed at the highest augmentation level.

### Validity of the overfitting limit estimate

**Figure 5:**
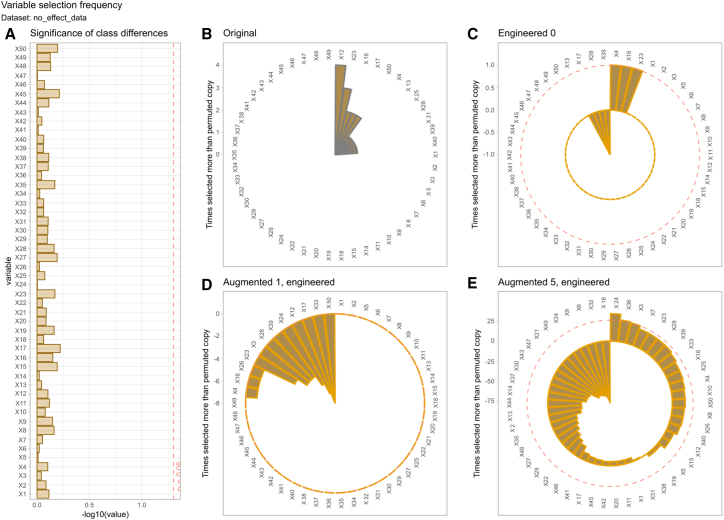
Control of false-positive feature selection in the absence of group differences using generative data augmentation The “no_effect_data” dataset was constructed such that both groups are statistically identical for all variables. Each variable is sampled from a normal distribution with the same mean and standard deviation across classes. (A) Displays the statistical significance of class differences for each variable, assessed by t-test and shown as -log_10_(p). (B–E) The circular bar plots display the frequency with which each variable was selected as “confirmed important” across 100 runs of the Boruta feature selection algorithm. Boruta uses a random forest classifier to compare the importance of the original variables to their permuted (engineered) counterparts. Selection frequencies are shown for the original data and for datasets augmented with one or five generated cases per original observation. A dashed line marks the bootstrapped 95^th^ percentile cutoff for the difference in selection frequency between the original and engineered variables. This cutoff serves as an overfitting control threshold. The variables are ordered according to the probability of an inflated alpha error.

Applying the AI-based generative data augmentation procedure to the “no_effect_data” dataset, in which both groups were sampled from normal distributions with identical means and standard deviations, provided a stringent test of the method’s robustness against spurious group effects. As shown in the bar plot of *p* values (Figure 5), all variables exhibited high *p* values far below significance level, confirming the absence of statistically significant differences between groups.

To capture the potential for error inflation due to overfitting, a 95^th^ quantile cutoff was used when comparing the selection of original and permuted variables. The correct observation in this dataset was no variable selected, i.e., none of the variables should cross the dashed line in Figures 5B–5E. This approach limited false-positive feature selection when data augmentation was used conservatively (i.e., generating one new data point per original observation). However, the results also indicate that increasing the proportion of generated data can lead to the inflation of group effects and the incorrect identification of more variables as important features. Starting with five new data points for each original, this occurred in two variables (“X24” and “X26”). In the present data, it was safe to add one new data point per original, i.e., doubling the sample size. However, adding five new instances per original crossed the limit, so adding fewer than five may be safe. These results underscore the importance of cautious data augmentation and validate the effectiveness of the overfitting limit estimate in controlling error rates in the absence of a true signal.

### Robust feature selection with moderate synthetic augmentation in preclinical lipidomics

**Figure 6:**
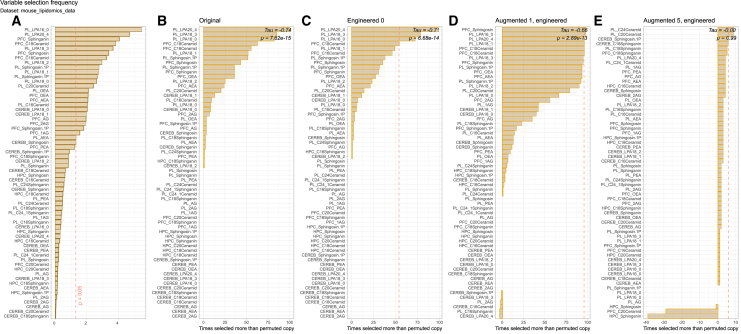
Variable selection frequency and statistical significance in the “mouse_lipidomics_data” dataset (A) The statistical significance of class differences for each lipid variable, assessed by ANOVA and shown as −*log*10(*p*). The dashed vertical line indicates the threshold for statistical significance (*p* = 0.05). (B–E) The frequency with which each variable was selected as important across different data regimes: the original dataset, engineered controls, and datasets augmented with increasing numbers of synthetic samples. Variables are ordered according to their significance. For each panel, the selection frequency reflects the proportion of times each variable was identified as important across repeated analyses. The overlaid statistical information shows the correlation between the frequency with which each variable is selected, corrected for the selection of each variable’s engineered counterpart, and its *p* value based on the above-mentioned ANOVA.

To assess the robustness of variable selection under data augmentation, the mouse lipidomics dataset (“mouse_lipidomics_data”) was analyzed, which includes quantitative profiles of 62 lipid mediators measured in plasma and three brain regions from 26 SJL/J mice across three experimental groups (Figure 6). Kendall’s tau rank correlation coefficients revealed strong negative correlations among the original (τ = −0.74, *p* = 7.6 × 10^−15^), engineered control (τ = −0.71, *p* = 6.7 × 10^−14^), and 1× augmented (τ = −0.66, *p* = 2.9 × 10^−13^) datasets. These results demonstrate that with moderate data augmentation (one synthetic sample per real sample), variables with greater statistical significance (lower *p* values) were preferentially selected as important. Notably, no overfitting effect was observed at this level of augmentation, and the relationship between statistical significance and variable selection remained robust. Furthermore, all of the top lipid mediators that were originally reported as the most significant (Lysophosphatidic Acid [LPA] 16:0 and 20:4 in plasma and sphingosine, sphinganine, and C18 ceramide in the prefrontal cortex) were consistently among the top-ranked features in the present analysis. Additional top hits also showed high statistical significance, suggesting that the variable selection procedure did not introduce false-positives. However, in the 5× augmented dataset, the correlation was essentially nonexistent (τ = −0.00, *p* = 0.99), suggesting a breakdown in the relationship between statistical significance and variable selection when data augmentation is excessive. This clear overfitting effect underscores the risk of generating excessive synthetic data, which can obscure true signals and compromise feature selection validity.

### Evaluating feature selection stability in diverse biomedical data

For each additional biomedical dataset, we evaluated the association between statistical significance and the frequency of variable selection under different data processing and augmentation methods. The key results, including the effects of feature engineering and varying levels of synthetic data augmentation, are summarized in Table 1.

**Table 1:** Kendall’s tau rank correlation coefficients (with *p* values in parentheses) are shown for each dataset under four data processing conditions: original (unmodified), engineered_0 (feature engineering or control processing), augmented_1_engineered (moderate augmentation: one synthetic sample per original case), and augmented_5_engineered (heavy augmentation: five synthetic samples per original case)

Dataset name	Original	Engineered_0	Augmented_1_engineered	Augmented_5_engineered	Conclusion
mouse_lipidomics_data	−0.735 (*p* = 7.62E-15)	−0.713 (*p* = 6.678E-14)	−0.661 (*p* = 2.89E-13)	−0.001 (*p* = 0.99013)	Augment moderately

**Preclinical 2**					

heart_failure_clinical_records	−0.869 (*p* = 0.00103)	−0.692 (*p* = 0.01059)	−0.539 (*p* = 0.03114)	NA	Augment moderately
Indian_liver_patient_data	−0.854 (*p* = 0.00064)	−0.841 (*p* = 0.00085)	−0.341 (*p* = 0.17621)	NA	Do not augment
pain_thresholds_sex_data	−0.853 (*p* = 0.00062)	−0.597 (*p* = 0.01662)	−0.697 (*p* = 0.003)	NA	Augment moderately
gallstone_data	−0.582 (*p* = 0.0000013)	−0.568 (*p* = 0.0000029)	−0.587 (*p* = 0.0000002)	0.015 (*p* = 0.9139)	Augment moderately
Golub_data	−0.62 (*p* = 1.08⋅10^−23^)	−0.61 (*p* = 1.75⋅10^−22^)	−0.6 (*p* = 2.18⋅10^−23^)	−0.47 (*p* = 3.66⋅10^−17^)	Augment moderately

Negative tau values indicate that variables with higher statistical significance were more frequently selected as important features. The Conclusion column summarizes the recommended augmentation strategy for each dataset based on the stability and significance of the correlation across conditions.

NA, the correlation could not be computed due to insufficient or uninformative data.

In the “heart_failure_clinical_records” dataset, strong negative correlations were observed between statistical significance and variable selection frequency in the original (τ = −0.87, *p* = 0.001), engineered (τ = −0.69, *p* = 0.011), and moderately augmented (τ = −0.54, *p* = 0.031) datasets. This indicates that statistically significant variables remained the most frequently selected features up to moderate augmentation. However, excessive augmentation resulted in a breakdown of this relationship, highlighting the risk of obscuring true signals with too much synthetic data.

For the “Indian_liver_patient” dataset, the error signal served as an effective stopping criterion. Although a strong negative correlation remained in both the original (τ = −0.85, *p* = 0.0006) and the engineered (τ = −0.84, *p* = 0.0008) datasets, the correlation weakened and became insignificant after moderate augmentation (τ = −0.34, *p* = 0.18). Further augmentation rendered the correlation uncomputable, indicating that synthetic data generation quickly undermines the interpretability of feature selection for this dataset, likely due to the arbitrary nature of its classification target.

In the “pain_thresholds_sex” dataset, a similarly strong negative correlation was found in the original data (τ = −0.85, *p* = 0.0006). This relationship remained robust in both the engineered (τ = −0.60, *p* = 0.017) and moderately augmented (τ = −0.70, *p* = 0.003) datasets. As with the other datasets, excessive augmentation disrupted the association, further emphasizing the importance of limiting synthetic data generation to preserve meaningful feature selection.

In the “gallstone” dataset, strong negative correlations between statistical significance and variable selection frequency were observed consistently across the original (τ = −0.58, *p* < 0.001), engineered control (τ = −0.57, *p* < 0.001), and moderately augmented (τ = −0.59, *p* < 0.001) datasets, all reflecting very large effect sizes. These results suggest that feature selection remained robust and aligned with statistical significance even after moderate augmentation. However, with heavy augmentation (five synthetic samples per original case), the correlation disappeared (τ = 0.01, *p* = 0.91).

In the “Golub_data” cancer genomics dataset, a strong negative correlation was observed in the original data (τ = −0.62, *p* < 0.001). This relationship remained robust in the engineered (τ = −0.61, *p* < 0.001) and moderately augmented (τ = −0.60, *p* < 0.001) datasets. However, excessive augmentation weakened the association (τ = −0.47, *p* < 0.001), highlighting the importance of limiting augmentation to preserve meaningful feature relationships, while the former results also demonstrate that moderate augmentation, specifically one synthetic sample per original data point, is safe.

Together, these results show that moderate data augmentation can maintain robust feature selection. This is true in various biomedical datasets. However, excessive augmentation can obscure true associations and should be approached with caution. Furthermore, some datasets clearly discourage augmentation, demonstrating that engineered features effectively signal potential risks. In these cases, conclusions drawn from augmented data may differ from those obtained from the original data, underscoring the importance of refraining from augmentation when indicated by the error signal.

## Discussion

### Context and risks of data augmentation

The use of AI for synthetic data generation addresses the persistent challenge of small sample sizes in preclinical and biomedical research, which often limits the translation of findings into clinical advances. AI-based data augmentation extends established techniques such as SMOTE and its derivatives, which have been shown to improve model fairness and predictive performance.^24^ Other than some simpler oversampling techniques or naive generation of data by adding arbitrary random noise, which may subject these techniques to the so-called curse of dimensionality,^25^ genESOM is based on a precise, neural network-based definition of neighborhood. This ensures that the data fit well into the existing structure without blurring it,^12^ as recalled in the example shown in Figure 2. However, all such methods entail the risk of amplifying noise and inducing overfitting.^7^

### Error inflation control with genESOM

The proposed method of implicit error inflation control via dimensionality modulation leverages specific properties of a generative AI that is based on emergent self-organizing maps (ESOM). Experiments with artificial datasets demonstrate that, within the genESOM framework and using random forest out-of-bag feature importance analysis, error inflation scales proportionally with effect size (as shown in the “ascending_significance_data” experiment). When data augmentation is applied within controlled limits, amplification of random noise remains below the threshold at which irrelevant variables are spuriously identified as important (as observed in the “no_effect_data” experiment). The goal of augmentation is to moderately increase sample size, not to create implausibly large expansions (e.g., *n* = 10 to *n* = 1,000), which would risk overfitting and inflated false-positives. This aligns with prior suggestions that calibrated augmentation can improve reproducibility.^20^

### Applications to biomedical datasets

Our results with biomedical data support these findings. With moderate augmentation (e.g., one synthetic data point per observed sample in the “mouse_lipidomics_data”), variable importance and statistical significance were robustly preserved. The implicit error signal within genESOM effectively detected when excessive augmentation (e.g., five synthetic cases per original) should be avoided, as this led to a loss of meaningful association between variable importance and original statistical significance. Additional biomedical examples further support these conclusions. For instance, with the “Indian_liver_patient” dataset, the framework generated a warning signal to avoid augmentation, as its structure was susceptible to inflated, irrelevant differences. Thus, by demonstrating both the limits to augmentation and the possibility of recommending no augmentation, this approach provides a practical solution to the problem of generative AI inflating random noise.

### Avoiding the enlargement of biased distributions in rare cases

The proposed algorithm cannot eliminate bias inherent in the original dataset; however, it prevents that bias from being magnified by augmentation. By coupling structure-aware sampling with diagnostic control features, it ensures that rare cases are not artificially over-represented unless they are biologically or statistically supported by the dataset’s intrinsic structure. The algorithm avoids disproportionately enlarging the effect of rare cases in biased distributions through (1) structure-based generation rather than random oversampling and (2) engineered negative controls as bias sentinels. Unlike naive oversampling, genESOM generates synthetic samples using neighborhoods defined by the intrinsic structure of the data. This means synthetic data are not created arbitrarily or disproportionately for rare cases but only within the boundaries of learned data neighborhoods. As a result, the algorithm respects the density of the data space and rare cases remain rare unless their structure justifies augmentation. Second, the algorithm injects permuted “noise variables” after the structural learning phase. These serve as built-in controls to monitor whether synthetic data begin to distort feature importance rankings. If augmentation inflates spurious signals, which could include over-amplification of rare or outlier cases, the diagnostic signal is triggered, warning the user to stop further augmentation.

### Biological plausibility

The approach shows a high degree of biological plausibility when applied under appropriate conditions. It maintains known pathophysiological signals in preclinical and clinical datasets, prevents the artificial inflation of noise in settings where no true differences exist, and explicitly warns against over-augmentation that would break mechanistic plausibility. At the same time, it remains sensitive to dataset quality, correctly discouraging augmentation where biological meaning is weak or unstable. This makes it a biologically credible tool for exploratory biomedical research, though not one intended for confirmatory trials or definitive biomarker discovery.

### Statistical significance versus predictive utility

While our workflow uses the association between variable importance and statistical significance to control for error inflation, it is crucial to clarify that these concepts are not interchangeable. As discussed in STAR Methods and supported by independent evidence,^26^ statistical significance and a variable’s predictive value are related but distinct. Statistical tests remain standard but rely on assumptions about data distributions that are often based on convention rather than direct evidence.^27^ For example, a non-significant result from the Kolmogorov-Smirnov test^28^ does not confirm identical distributions, but rather insufficient evidence against the null hypothesis. The choice of statistical test can influence variable rankings, and statistical significance may diverge from predictive utility, especially in settings where classical methods are structurally limited.^29^ In such cases, predictive performance, measured through prospective validation, becomes more relevant, especially for generalizability. Increasing sample size with genESOM enables the application of machine learning to small datasets, but caution is required: data should be split into training and validation subsets before augmentation, and augmentation should be performed separately for each.

### Translational integration and ethical safeguards

The genESOM-based approach could be incorporated into biomedical research pipelines at the stage of exploratory data analysis, particularly when sample sizes are small due to ethical, practical, or financial constraints. By modestly augmenting datasets after quality control but before downstream modeling, researchers can stabilize feature rankings and improve the robustness of hypothesis generation. The built-in error signal serves as a safeguard, indicating when augmentation remains valid and when it risks obscuring true biology. In translational contexts, such as preclinical drug discovery or early clinical studies, this approach may help prioritize candidate biomarkers or predictors. However, its role should remain confined to exploratory analyses; confirmatory studies and regulatory submissions require real data only. Importantly, the method does not remove biases inherent in the original data but prevents them from being artificially amplified through augmentation. By coupling structure-aware sampling with diagnostic controls, rare cases are not overrepresented unless this is biologically or statistically justified by the dataset’s intrinsic structure. Synthetic data are generated within learned data neighborhoods, ensuring that rare cases remain rare unless the structure itself warrants their reinforcement.

Adoption of genAI also brings ethical and regulatory challenges.^30^ Augmentation may replicate or amplify biases present in the original data, so demographic balance and fairness must be monitored. Privacy risks persist if generative models overfit small cohorts, requiring protective measures like strict access control. Most importantly, patient safety demands that synthetic augmentation not be misapplied to clinical decision-making. To safeguard against misrepresentation, genESOM incorporates engineered negative controls (permuted noise variables) after the structural learning phase. These act as diagnostic sentinels: if augmentation begins to distort feature importance rankings or inflate spurious signals, including exaggerated effects of rare or outlier cases, the diagnostic signal warns the user to stop further augmentation. Clear labeling of augmented datasets and transparent reporting of stopping criteria will be key for responsible use.

### Cross-domain evidence for structural preservation in safe data augmentation

Recent studies highlight the importance of preserving intrinsic data structures, both statistical and temporal, during simulation and augmentation. Maintaining temporal correlations among environmental risk factors, such as particulate matter, ammonia, and humidity, was shown to be crucial for accurate modeling of COVID-19 severity in Delhi.^31^ Building on this, it was shown that spatiotemporal dependencies between air pollutants and infection dynamics must be conserved to ensure valid causal inference.^32^ These findings parallel the present study’s emphasis on maintaining variable significance and ordering during generative augmentation: just as disrupting temporal or spatial correlation can distort environmental risk modeling, uncontrolled synthetic expansion can inflate false associations in biomedical data.

### Potential benefits and pitfalls

This work forms part of a broader research program aiming to enhance predictivity in preclinical studies with limited sample sizes (Figure 1). The foundational genESOM method and its comparison to alternative generative AI approaches were described previously.^12^ The present study extends this by integrating error control signals to mitigate synthetic data error inflation, an essential step for reliable data augmentation. Together, these efforts provide a phased framework for responsible and effective deployment of generative AI in biomedical research.

Dataset augmentation presents both opportunities and risks within biomedical AI. When combined with rigorous error monitoring and structural awareness, it can enhance data representativeness and statistical robustness, as highlighted recently.^33^ Conversely, unregulated augmentation may degrade data quality, inflate false discoveries, and undermine confidence in AI-driven medical insights. The future of biomedical AI should, therefore, emphasize the generation of high-quality, structure-preserving, and ethically sound data rather than merely increasing dataset sizes.

Increasingly, the role of data quality in AI applications is being emphasized.^34^ High-quality data underpins trustworthy AI applications in medical research. The effectiveness of any AI model is contingent upon the accuracy, representativeness, and completeness of the training data,^35^ stressing the importance of high-quality data.^36^ Dataset augmentation involves generating new samples through transformations of existing data or generative modeling, which can mitigate class imbalance, improve model robustness, and enhance statistical reliability.^37^ For instance, careful preprocessing and data balancing can boost model performance, as demonstrated in breast cancer survivability analysis.^38^

However, techniques like resampling may inadvertently reduce model accuracy on external validation data, highlighting the delicate trade-off between quantity and quality.^39^ Moreover, careful consideration is necessary regarding the representativeness of the dataset. For example, suppose a complete dataset capturing a problem consists of 70 red, 25 blue, and 5 yellow data points. However, suppose an experiment samples only 10 data points and only sees red and blue. In this case, augmentation using genESOM would not compensate for the lack of yellow samples, which could be captured with a larger sample size.

In biomedical contexts, where datasets encode subtle physiological relationships, generating artificial data risks distorting the underlying biological signals.^33^ Addressing data quality directly influences the reliability and performance of AI.^34^ Our generative AI framework is designed to mitigate error inflation during data augmentation. We demonstrate that although generative models can preserve structure, excessive generation can inflate type I error rates. Validation across artificial and biomedical datasets shows that moderate augmentation, i.e., approximately one synthetic sample for each original, can effectively double sample size without compromising feature ranking stability or statistical validity. In contrast, excessive augmentation disrupts the relationship between variable importance and statistical significance, leading to unreliable feature selection and spurious associations.^33^

As the landscape of healthcare evolves with AI, data quality and robust preprocessing methods remain critical for enhancing the reliability and generalizability of AI applications. Addressing these challenges will reshape modern healthcare, enabling data-driven decision-making and precision medicine. Overall, the findings of this report underscore the practice of considering representativeness in dataset augmentation and highlight the need for ongoing attention to data quality and structure in AI applications.

### Limitations of the study

The present data augmentation method was developed with small preclinical or clinical numerical tabular datasets in mind. However, obtaining a reliable and robust threshold for error detection using bootstrapping remains challenging, particularly in settings with few samples but a very large number of variables. This limits applicability in high-dimensional datasets, where computing power can also become a constraint. Image, genetics, and time-series datasets are likewise not directly supported, since in the present framework they require transformation into tabular form through feature detection and extraction. For example, histological images would need to be converted into quantitative features, such as cellular subtype counts, before analysis. These restrictions stem not only from the inherent complexity of such data but also from a fundamental limitation of the underlying genESOM method, which is constrained to static, tabular input and cannot incorporate temporal information. As a result, direct processing of raw images, sequences, or time-series data falls outside the scope of this framework. Importantly, the framework is not designed for image generation per se; applications such as direct synthesis of medical images or generation of time-series data are more appropriately addressed by generative adversarial networks.^40^^,^^41^ While in principle our method might be adaptable to such data, it was not developed or tested for these purposes. Instead, this work emphasizes small-sample tabular datasets where generating additional data points is often impossible or prohibitively costly. Finally, the present genAI framework multiplies the existing number of cases and can only produce integer multiples of the original dataset size. If a specific target size does not match such multiples, the dataset must be downsampled accordingly.

The methodological framework itself is model agnostic. A limitation of this study, though not of the framework, is that we assessed performance using only random forest-derived feature importance. This choice was deliberate, as random forests have consistently demonstrated suitability for small and noisy tabular biomedical datasets. While alternative feature importance measures or ensemble approaches like Mixture of Experts could enhance robustness, especially in large-scale omics applications,^42^^,^^43^ these extensions are beyond the scope of this initial work focusing on framework establishment and validation.

The proposed error control signal is specific to genESOM and relies on its unique ability to accommodate dimensional changes between structure detection and data generation. At present, this mechanism cannot be directly transferred to other generative algorithms, so comparative evaluation against broader classes of generative models lies beyond this study’s scope, which centers on introducing and validating the genESOM-specific signal.

### Conclusion

Preclinical and many human studies frequently rely on modest sample sizes,^44^ although simulations suggest adequate sizes are often about double those typically used.^45^ While data augmentation with early stopping is reasonable, avoiding error inflation and amplification of irrelevant differences is essential. This framework leverages genESOM’s unique ability to modulate data dimensionality between structure learning and data generation, a feature uncommon in other generative algorithms.^12^ Validation on artificial and biomedical datasets shows that moderate augmentation preserves variable importance and maintains strong negative Kendall’s tau correlations between statistical significance and feature importance, reflecting robust feature selection across diverse datasets including preclinical lipidomics and clinical data. Experiments consistently showed that generating one synthetic data point per original is safe, enabling, for example, reductions in laboratory animal use by half. The integrated error signal reliably raises a red flag when augmentation is not advisable, fulfilling genAI’s potential. However, practical adoption is primarily targeted at numerical tabular data without time-series or imaging complexities, and further limitations are noted above.

For strictly confirmatory experiments, however, we maintain that generative data augmentation remains unacceptable, consistent with previous findings from simpler oversampling approaches.^20^ However, for non-confirmatory studies, our framework offers a robust means to enhance reproducibility without compromising validity, which again agrees with independent conclusions.^20^ Our validation focused on genESOM due to the specificity of the error detection method, though previous benchmarking has demonstrated its competitive generative performance. In summary, this framework provides a practical solution for improving knowledge extraction from preclinical studies, particularly in exploratory contexts characterized by limited statistical power and an elevated risk of error inflation.

## Resource availability

### Lead contact

Requests for further information and resources should be directed to and will be fulfilled by the lead contact, Jörn Lötsch (j.loetsch@em.uni-frankfurt.de).

### Materials availability

This study did not generate any new unique materials.

### Data and code availability

• All the source data used in this analysis are listed in the key resources table. This paper analyzes existing, publicly available data, accessible at the following DOIs: mouse_lipidomics_data: Mendeley Data: https://doi.org/10.17632/m2p6rr9v36.1, heart_failure_clinical_records: UC Irvine Machine Learning Repository: https://doi.org/10.24432/C5Z89R, Indian_liver_patient_data: UC Irvine Machine Learning Repository: https://doi.org/10.24432/C5D02C, pain_thresholds_sex_data: Mendeley Data: https://doi.org/10.17632/9v8ndhctvz.1, gallstone_data: UC Irvine Machine Learning Repository: https://doi.org/10.1097/MD.0000000000037258, and Golub_data: Bioconductor: https://doi.org/10.18129/B9.bioc.golubEsets. Further descriptions of the datasets can be found in the STAR Methods section.
• All original code has been deposited at GitHub: https://github.com/JornLotsch/genESOMerrorSignal and is publicly available at https://doi.org/10.5281/zenodo.17639886 as of the date of publication.
• Any additional information required to reanalyze the data reported in this paper is available from the lead contact upon request.

## Acknowledgments

J.L. was supported by the 10.13039/501100001659Deutsche Forschungsgemeinschaft (DFG Lo 612/16-1).

## Author contributions

J.L., conceptualization of the project, developing the project, theoretical background, programming, performing experiments, writing of the manuscript, data analyses and creation of the figures, funding acquisition, and revision of the manuscript. A.H., theoretical background, critical check of scientific content, and revision of the manuscript. D.K., dataset research, selection and retrieval, evaluation of the feasibility of the approach for biomedical researchers, contributing to the writing of the manuscript, and revision of the manuscript.

## Declaration of interests

The authors declare no competing interests.

## Declaration of generative AI and AI-assisted technologies in the writing process

During the preparation of this work the authors used the jetbrains AI Assistant plugin (version 251.26094.80.19, https://plugins.jetbrains.com/plugin/22282-jetbrains-ai-assistant) to refactor and comment code. After using it, the authors reviewed and edited the content as needed and take full responsibility for the content of the publication.

## STAR★Methods

### Key resources table

**Table:** 

REAGENT or RESOURCE	SOURCE	IDENTIFIER
**Deposited data**		

mouse_lipidomics_data	Schmitz et al.,^46^ de Bruin et al.^47^	https://doi.org/10.17632/m2p6rr9v36.1 (https://data.mendeley.com/datasets/m2p6rr9v36/1)
heart_failure_clinical_records	Chicco et al.^48^	https://doi.org/10.24432/C5Z89R (https://archive.ics.uci.edu/dataset/519/heart+failure+clinical+records)
Indian_liver_patient_data	Straw et al.^49^	https://doi.org/10.24432/C5D02C (https://archive.ics.uci.edu/dataset/225/ilpd+indian+liver+patient+dataset)
pain_thresholds_sex_data	Lötsch et al.,^50^ Doehring et al.^51^	https://doi.org/10.17632/9v8ndhctvz.1 (https://data.mendeley.com/datasets/9v8ndhctvz/1)
gallstone_data	Esen et al.^52^	https://doi.org/10.1097/md.0000000000037258 (https://archive.ics.uci.edu/dataset/1150/gallstone-1)
Golub_data	Golub et al.^53^^,^^54^	https://doi.org/10.18129/B9.bioc.golubEsets (https://bioconductor.org/packages/golubEsets)

**Software and algorithms**		

R Version 4.5.1 (used for statistical analysis)	R core team^55^	https://cran.r-project.org
R package “Umatrix”	R package provided by the authors^12^^,^^17^	https://cran.r-project.org/package=Umatrix
PyCharm integrated development environment (version 2025.1.2) Professional Edition (used for coding)	JetBrains, Prague, Czech Republic	https://www.jetbrains.com/pycharm/
PyCharm AI Assistant plugin (version 251.26094.80.19) (used to facilitate coding)	JetBrains, Prague, Czech Republic	https://plugins.jetbrains.com/plugin/22282-jetbrains-ai-assistant
Python (version 3.11.7 for Linux) (used for plotting)	Python Software Foundation^56^	https://www.python.org/
Code for this project	This article	https://doi.org/10.5281/zenodo.17639886 (https://github.com/JornLotsch/genESOMerrorSignal)

**Other**		

ascending_significance_data	Associated GitHub repository provided by the authors	https://doi.org/10.5281/zenodo.17639886 (https://github.com/JornLotsch/genESOMerrorSignal/blob/main/generate_artifical_datasets.R)
no_effect_data	Associated GitHub repository provided by the authors	https://doi.org/10.5281/zenodo.17639886 (https://github.com/JornLotsch/genESOMerrorSignal/blob/main/generate_artifical_datasets.R)

### Method details

#### Computational setup

The coding to validate the error inflation control method was primarily done in R (version 4.5.1 for Linux)^55^^,^^57^ using the PyCharm integrated development environment (version 2025.1.2 Professional Edition, JetBrains, Prague, Czech Republic) with the AI Assistant plugin (version 251.26094.80.19, https://plugins.jetbrains.com/plugin/22282-jetbrains-ai-assistant). The computations were performed on an Intel® Core™ i7-13700H notebook (Intel Corporation, Santa Clara, CA, USA), running on Ubuntu Linux 24.04.3 LTS (Canonical, London, UK). Additional components were implemented in Python (version 3.11.7 for Linux),^56^ which is freely available at https://www.python.org/.

#### Summary of the generative AI used (genESOM)

The following paragraphs summarize the key functions of ESOM^13^^,^^14^ and its generative extension genESOM.^12^

##### ESOM: Neighborhood-preserving modeling of distance- and density-based data structures

The generative AI method addressed here^12^ is based on so-called “emergent” self-organizing maps (ESOM) of artificial neurons,^13^^,^^58^ a neural network that extends the classical self-organizing map (SOM) algorithm.^59^ ESOM projects high-dimensional data onto a large two-dimensional grid consisting of many (e.g., a few thousand) artificial neurons while preserving neighborhood relationships between data points. The emergent components are realized as U- and P-matrices, which detect and model, respectively, complex distance- and density-based structures in high-dimensional data. Comparisons with other unsupervised structure detection algorithms consistently demonstrate that this method rivals or outperforms other unsupervised methods, including clustering algorithms, autoencoders, and manifold learning projection methods. Due to its capability as a non-parametric approach to validly modeling relevant class-related structures of a given dataset, it can therefore serve as a basis for data generation.

##### ESOM based modeling of distance-based data structures

The method has been described in detail previously.^12^ In brief, the ESOM learning process is governed by the following update rule for each neuron’s weight vector:

(Equation 5) $$\Delta w_{i}=\eta \left(t\right)\cdot h\left({BMU}_{i},r,t\right)\cdot \left(x_{i}-w_{i}\right)$$

where *x*_*i*_ is a data point, *BMU*_*i*_ is the best matching unit (closest neuron), *h*(·) denotes the neighborhood function with the radius parameter *r* described below, and *η*(*t*) is the learning rate, both of which decrease over time.^60^

After training, the U-matrix is computed to visualize the average high-dimensional distances between neighboring neuron prototypes, highlighting potential cluster boundaries.^58^^,^^61^

##### ESOM based modeling of density-based data structures

Based on the generation of the U-matrix, a P-matrix can be constructed to estimate density-based structures in the data^13^ as a further component for possible consideration in data augmentation As previously described,^12^ this process starts from the trained U-matrix, i.e., at a point where the neurons reflect the structure of a dataset. For the P-matrix, the density *p*(*n*_*i*_) for each neuron *n*_*i*_ is defined as the number of data points within a hypersphere of radius *r* around its prototype vector:

(Equation 6) $$p\left(n_{i}\right)=\left|\left\{x:d\left(x,w\left(n_{i}\right)\right)\leq r\right\}\right|$$

The critical radius, *r*, is determined by modeling the distribution of U-matrix heights, or distances between neurons, using a bimodal Gaussian mixture model (GMM). This model is fitted using the expectation maximization (EM) algorithm.^62^ The resulting fit provides an appropriate radius for robust density estimation.^58^ Examples of the effects of over or underestimating the radius are given in.^12^

##### ESOM based characterization of local neighborhoods

The radius *r* defines the local neighborhood. That is, the neighborhood of a data point *x*_*i*_ is quantified by the probability that another point, *x*_*j*_, at a distance *d*(*x*_*i*_,*x*_*j*_) from *x*_*i*_, belongs to the neighborhood. This probability is calculated using a sigmoid-based function.

(Equation 7) $$p_{ij}=1-\frac{1}{1+e^{-\frac{10\cdot d\left(x_{i},x_{j}\right)}{c-1}}}$$

where *p*_*ij*_ is the probability that *x*_*j*_ is in the neighborhood of *x*_*i*_, *d*(*x*_*i*_,*x*_*j*_) is their distance, and *c* is a scaling constant that sets the effective size of the neighborhood.

##### genESOM: Generative capability of ESOM

The genESOM data augmentation pipeline has two main steps. First, the critical radius parameter is established based on a structural investigation of the dataset. Next, new data samples are generated by applying a specified probability distribution to samples within the neighborhood defined by the critical radius around existing data points.

##### Estimation of the critical parameter for data generation

Related to the above radius *r*, the neighborhood-preserving property of the ESOM is used to generate new data. After constructing the U- and P-matrices, the next key step is determining the generative parameter, specifically the radius *r* of the hyperspheres centered on each original data point. This radius defines the local neighborhood from which synthetic data is sampled. The optimal radius *r* is derived from the distribution of distances in the data, particularly using the abstract U-matrix heights (*AU*_*heights*) and a Gaussian mixture model to identify the Bayesian decision boundary *t*_*AU*_ between clusters.

A central aspect of genESOM is the structure-based estimation of the *r* parameter, which ensures that the generation process is informed by the data’s intrinsic organization. This approach contrasts with oversampling methods that rely on arbitrary jitter and do not account for the actual data structure. By contrast, using a data-driven radius allows genESOM to avoid two major pitfalls associated with generating high-dimensional data.^12^ First, it prevents the artificial concentration of generated points on the surface of a hypersphere around the seed.^25^ Second, it maintains the integrity of neighborhood boundaries, minimizing the risk of synthetic points crossing into unrelated regions and preserving the true local structure of the data.^63^

##### Augmenting data sets based on learned inherent structures

To capture dense and sparse regions, the algorithm generates *k* synthetic points for each data point. Eighty-five percent of these points are sampled from within the core neighborhood (*p*_*ij*_>0.95), 15% from intermediate distances (0.10<*p*_*ij*_<0.95), and 5% from the outer region ($\frac{d\left(x_{i},x_{j}\right)}{c}\leq 2$). Unlike most other generative AI approaches, genESOM has the unique property of being able to explicitly change the dimensionality of synthetic data during the generative step. This allows genESOM to adapt its output to specific analytical needs or downstream applications while preserving the original intrinsic structure of the data set and the boundaries between classes or clusters.

#### Assessment of feature importance using the Boruta algorithm

In principle, the present method is model-agnostic in regard to the feature importance algorithm; however, the genAI model must allow dimensionality changes as genESOM. For the present evaluations, machine learning approaches were chosen, specifically random forest classifiers,^64^ which have demonstrated superior performance compared to other supervised methods in recent studies.^65^ Random forests inherently provide estimates of feature importance through permutation weighting of out-of-bag (OOB) samples, i.e., samples not used during training, by measuring the decrease in classification accuracy when a feature’s values are randomly permuted.^64^ This approach is particularly effective for tabular numerical data, often outperforming deep learning neural networks^66^^,^^67^ and logistic regression.^68^

To facilitate robustness, the Boruta algorithm is used as a wrapper for random forests.^23^ Boruta uses permutation importance and performs a top-down search for relevant features. This makes it preferable to simple permutation importance for stabilizing feature selection in high-dimensional settings. In each iteration, Boruta adds “shadow” features to the dataset. These are randomly permuted copies of the original variables. Then, a random forest is trained on the full dataset, and feature importance is quantified using the out-of-bag (OOB) error estimation intrinsic to the random forest algorithm. Each tree in the random forest is trained on a bootstrap sample of the data, with approximately one-third of the observations left out (the OOB samples) for that tree. Importantly, Boruta uses a statistical significance criterion based on “shadow” features, making it consistent with the statistical significance criterion used in the present framework for evaluating the agreement of the results with the original statistical significance criteria.

The prediction accuracy of the tree is then evaluated on its respective OOB samples. To assess a feature’s importance, its values are randomly permuted in the OOB samples, and the resulting decrease in prediction accuracy (or increase in error) is measured. This process is repeated for each feature and tree, and the average decrease in accuracy across all trees is used as the feature’s importance score. Importantly, the importance of each feature is evaluated using data that was not seen by the tree during training (the OOB samples), not the data used to train the tree. This provides an unbiased estimate of variable importance that does not rely on a separate validation set or explicit cross-validation. Boruta compares the importance of each original feature to the maximum importance achieved by any shadow feature. Features are then categorized as “confirmed” significant, “tentatively” significant, or “rejected” non-significant based on whether their importance is significantly higher than that of the shadow features, thereby circumventing the need to impose an arbitrary threshold for feature importance.

### Quantification and statistical analysis

All statistical analyses were performed on either artificially generated datasets with known ground truth or on publicly available biomedical datasets. Statistical details for each analysis are reported in the figure labels. Measures of center and variability are reported as mean ± standard deviation (SD). Statistical significance was defined as *p* < 0.05 unless otherwise noted. Values and meaning of n for each data set can be found in the “data sets” section. Used statistical tests can be found below and all prerequisites were checked.

#### Experimentation

To validate the error inflation control method, we first employed artificial datasets specifically designed to reflect known ground truths regarding variable importance. We then validated the method using several publicly available biomedical datasets from preclinical and clinical research.

##### Feature importance ranking

One of the objectives of the validation experiments was to reproduce the original feature importance rankings or confirm the non-informativeness of the variables when the dataset was augmented using modest data generation via genESOM. These experiments followed the stepwise approach described above to generate new data points.

##### Agreement with statistical significance ranking

A further objective was to assess the agreement of the above feature ranking with the ranking based on the statistical significance of group differences. To assess the association between the statistical significance of each lipid variable and its feature importance, Kendall’s tau rank correlation coefficient was computed. Specifically, for each variable, we calculated the p-value from analyses of variance (ANOVA) in the case of more than two classes or study groups, or from t-tests^69^ in the two-class case. As described above, feature importance was quantified within our methodological framework as the frequency with which the true variable was selected over its engineered counterpart across 100 Boruta feature selection runs. The Kendall’s tau coefficient was then used as a non-parametric statistic to quantify the strength and direction of the monotonic relationship between p-values and feature importance, as it does not assume normality or linearity in the data.^70^ A negative correlation indicates that variables with lower p-values (greater statistical significance) tend to have higher feature importance, as hypothesized.

#### Implementation

The validation tasks were primarily run in R, and the custom code is available at https://github.com/JornLotsch/genESOMerrorSignal. The analysis used several R libraries. The “Umatrix” R library (https://CRAN.R-project.org/package=Umatrix^17^) was used to compute and visualize emergent self-organizing maps. The “dbt.DataIO” library (https://github.com/Mthrun/dbt.DataIO) handled the input and output of the data for this analysis. Feature importance analysis was conducted using the “Boruta” library (https://CRAN.R-project.org/package=Boruta^23^). The “caret” library was applied to assess classification performance (https://cran.r-project.org/package=caret^71^), and the “reshape2” library provided internal functions for data manipulation (https://cran.r-project.org/package=reshape2^72^). The “ggplot2” library (https://CRAN.R-project.org/package=ggplot2^73^) was used for visualization, and figures were combined using the “cowplot” library (https://CRAN.R-project.org/package=cowplot^74^). Figure coloring utilized the colorblind-friendly “colorblind_pal” palette from the “ggthemes” R package (https://cran.r-project.org/package=ggthemes^75^). The “parallel” (https://CRAN.R-project.org/package=parallel^55^) and “pbmcapply” (https://CRAN.R-project.org/package=pbmcapply^76^) libraries managed parallel processing.

#### Data sets

Artificial datasets were generated to establish well-defined ground truths regarding variable importance. The method was further applied to five publicly available biomedical datasets derived from previously published preclinical and clinical research.

##### Artificial data sets

The generation of the artificial data sets is accessible via the R code at https://github.com/JornLotsch/genESOMerrorSignal/blob/main/generate_artifical_datasets.R.

##### Ascending statistical significance of class differences

An artificial dataset was constructed to provide a controlled test environment with known, systematically varying group differences (“ascending_significance_data”). The dataset consists of *n*_*v*_ = 50 variables, each measured across two classes with *n* observations per class (such as *n* = 10), resulting in a total of 2*n* observations per variable.

For each variable *i*, the observations in class 1 are drawn from a normal distribution *N*(*μ*_1,*i*_,1), and those in class 2 from a normal distribution *N*(*μ*_2,*i*_,1), where the standard deviation is fixed at 1 for all variables. The mean values are defined as: $\mu_{1,i}=1+\frac{\left(i-1\right)\cdot \left(\max_{1}-1\right)}{n_{v}-1}$ for class 1 and $\mu_{2,i}=1+\frac{\left(i-1\right)\cdot \left(\max_{2}-1\right)}{n_{v}-1}$ for class 2, where *max*_1_ = 50 and *max*_2_ = 40. This ensures that the difference between class means increases systematically across variables.

This design yields a dataset in which the statistical significance of class differences gradually increases from the first to the last variable. Thus, it is an ideal framework for evaluating whether the tested method amplifies errors proportionally to the underlying true differences rather than randomly or indiscriminately. The design ensures that each variable exhibits consistent and predictable differences between the two classes, with these differences growing larger across the dataset. Using this dataset allows one to rigorously assess the method’s ability to detect and control error inflation in the presence of a clear, graded signal.

##### Absence of statistical significance of class differences

To evaluate the robustness of the method against the inflation of negligible group differences, a second artificial dataset was created. In this dataset, all variables were sampled from distributions with identical means and standard deviations across the two classes. This dataset, called “no_effect_data”, contains *n*_*v*_ = 50 variables, each measured across two classes with *n* = 10 observations per class, resulting in a total of 2*n* observations per variable.

For each variable *i*, the mean *μ*_*i*_ was sampled from a discrete uniform distribution *Uniform*(10,30), and the standard deviation *σ*_*i*_ was sampled from a discrete uniform distribution *Uniform*(1,3) (in steps of 0.01). Each class was then populated with *n* observations randomly drawn from the same normal distribution *N*(*μ*_*i*_,*σ*_*i*_). This ensures that, for every variable, the two classes are sampled from identical distributions: *x*_1,*i*,*j*_ ∼ *N*(*μ*_*i*_,*σ*_*i*_),*j* = 1, …,*n* and *x*_2,*i*,*j*_ ∼ *N*(*μ*_*i*_,*σ*_*i*_),*j* = 1, …,*n*, where *x*_1,*i*,*j*_ and *x*_2,*i*,*j*_ denote the j^th^ observation of variable *i* in classes 1 and 2, respectively. Although the means and standard deviations may differ across variables, within each variable, the two classes are statistically indistinguishable. To further force the absence of group effects, two-sample t-tests were performed for each variable, and only those with a p-value above the predefined threshold of p ≥ 0.6 were included in the final dataset. From this subset, *n*_*v*_ variables were randomly selected for subsequent analyses. This design provides a stringent test of the method’s ability to avoid false detection of group differences in the absence of any true signal.

##### Biomedical data sets

To evaluate the method across diverse biomedical contexts, both in-house and publicly available datasets were analyzed. The in-house dataset comprises mouse lipidomics profiles, and four additional datasets were obtained from open-access biomedical repositories. Key descriptive information about the datasets is shown in the key resources table in the STAR Methods section.

#### Preclinical mouse lipidomics dataset

A real-world preclinical dataset was obtained from in-house preclinical research.^46^ The “mouse_lipidomics_data” dataset, provided at https://data.mendeley.com/datasets/m2p6rr9v36/1 along with a detailed description in,^77^ contains quantitative lipidomics profiles from 26 eight-week-old female SJL/J mice divided into three groups: a control group (n = 10), an experimental autoimmune encephalomyelitis (EAE) model group (n = 8), and an EAE model group treated with fingolimod (n = 8). For each animal, the concentrations of lipid mediators, including lysophosphatidic acids, ceramides, sphingolipids, and endocannabinoids, were measured in plasma and three brain regions (the cerebellum, hippocampus, and prefrontal cortex) using targeted liquid chromatography-tandem mass spectrometry. This finally yielded 62 variables containing lipidomic information. The original report^46^ highlighted five lipid mediators as the most significant: lysophosphatidic acid 16:0 and 20:4 in plasma and sphingosine, sphinganine, and C18 ceramide in the prefrontal cortex. These variables were central to the observed effects of fingolimod treatment on lipid signaling in the EAE model of multiple sclerosis.

#### Clinical datasets

In addition to an in-house human experimental data set, three further clinical data sets were retrieved from publicly accessible repositories.

The “heart_failure_clinical_records” data set was obtained from the UCI Machine Learning Repository (https://archive.ics.uci.edu/dataset/519/heart+failure+clinical+records). The dataset includes demographic, clinical, and laboratory data from patients with a history of heart failure who were classified as NYHA (New York Heart Association) class III or IV. The patients were between 40 and 95 years old, and the dataset includes information on sex (105 women and 194 men), smoking status, diabetes, anemia, blood pressure, serum creatinine, serum sodium, platelets, creatinine phosphokinase, and ejection fraction.^48^ The primary aim was to predict patient survival and identify the most informative clinical features using machine learning. Serum creatinine and ejection fraction emerged as the two most important predictors of survival. Models trained on these two features outperformed models using all variables. For instance, a random forest classifier using only these two features achieved a Matthews correlation coefficient (MCC)^78^ of +0.418 and an area under the curve (AUC) of 0.698, surpassing models trained on the full dataset. These findings, confirmed by statistical tests and alternative feature ranking methods, suggest that a minimalist feature set can provide robust survival predictions. Presently, only complete cases (203 survivors, 96 deaths) were included.

The “Indian_liver_patient” dataset was obtained from the UCI Machine Learning Repository (https://archive.ics.uci.edu/dataset/225/ilpd+indian+liver+patient+dataset). The dataset contains demographic and biochemical data from 583 patients (441 men and 142 women) collected in NorthEast Andhra Pradesh, India. Each record includes information on age (ranging from 4 to 90 years), gender, and nine laboratory measurements: total and direct bilirubin, alkaline phosphatase, alanine aminotransferase aspartate aminotransferase, total proteins, albumin, and the albumin/globulin ratio. The primary aim of the original study was to predict the presence of liver disease (416 cases, 167 controls) and to investigate potential sex-based disparities in predictive model performance.^49^ Presently, only complete cases (414 patients, 165 controls) were included.

The used in-house “pain_thresholds_sex” dataset (https://data.mendeley.com/datasets/9v8ndhctvz/1https://data.mendeley.com/drafts/9v8ndhctvz; publication of detailed description pending) includes quantitative sensory data from 125 healthy, unrelated, Caucasian adults (69 men and 56 women, aged 18–46 years, with a mean age of 25 ± 4.4 years). Participants underwent standardized assessments of pain thresholds in response to thermal (heat and cold), mechanical (blunt and punctate), and electrical stimuli in both non-sensitized and sensitized areas of the forearm. Pain thresholds were defined as the minimum stimulus intensity at which pain was first perceived. Stimulus intensity was measured in the appropriate physical units (e.g., °C for thermal, grams or Newtons for mechanical, and mA for electrical). The sensitization procedures involved the topical application of capsaicin for mechanical and heat stimuli or menthol for cold stimuli to induce localized changes in skin sensitivity. Each modality was tested in multiple repeated trials, and median or mean values were used as summary measures. The dataset contains 12 variables, including biological sex as the target variable,^50^ which was reused as target in the present report. Full methodological details, including stimulus devices, protocols, and sensitization procedures, are available in the original publication.^51^ This dataset supports investigations into individual variability in pain thresholds and the modulatory effects of sensitization across different stimulus modalities.

The “gallstone” dataset,^52^ again obtained from the UCI Machine Learning Repository at https://archive.ics.uci.edu/dataset/1150/gallstone-1, presents a machine learning-based approach for early prediction of gallstone disease using a combination of bioimpedance analysis (BIA) and laboratory data. It contains data from 319 individuals (161 with gallstones, 158 healthy controls), collected at Ankara VM Medical Park Hospital between June 2022 and June 2023. The dataset comprises 38 variables, covering demographic characteristics, comorbidities, detailed body composition metrics derived from BIA, and a range of laboratory blood markers. Gradient Boosting achieved the highest predictive accuracy (85.42%) and identified vitamin D, C-reactive protein, total body water, and lean mass as the most important predictors. This study demonstrates that machine learning models utilizing bioimpedance and laboratory data can offer an effective, noninvasive, and cost-efficient alternative for early gallstone detection, particularly in settings where access to imaging is limited.

Finally, the cancer genetics “Golub_data” dataset^53^^,^^54^ was used to cover genomics data. This dataset is available as sample data in the R package “golubEsets” (https://bioconductor.org/packages/golubEsets). The dataset comprises gene expression data from Affymetrix Hgu6800 microarrays of bone marrow samples from 72 patients diagnosed with acute leukemia. Of these patients, 47 had acute lymphoblastic leukemia (ALL) and 25 had acute myeloid leukemia (AML). The original dataset contains expression profiles for 7,129 genes. For this study, the top 150 genes, sorted by decreasing variance, were selected based on preprocessing conventions (https://rstudio-pubs-static.s3.amazonaws.com/3773_0afaead59a02436889abc68753e6c20a.html).
